# The impact of Roux-en-Y gastric bypass surgery on normal metabolism in a porcine model

**DOI:** 10.1371/journal.pone.0173137

**Published:** 2017-03-03

**Authors:** Andreas Lindqvist, Mikael Ekelund, Eliana Garcia-Vaz, Marcus Ståhlman, Stefan Pierzynowski, Maria F. Gomez, Jens F. Rehfeld, Leif Groop, Jan Hedenbro, Nils Wierup, Peter Spégel

**Affiliations:** 1 Neuroendocrine Cell Biology, Department of Clinical Sciences Malmö, Lund University Diabetes Centre, Malmö, Sweden; 2 Department of Surgery, Department of Clinical Sciences Malmö, Lund University, Lund, Sweden; 3 Vascular ET-coupling, Department of Clinical Sciences Malmö, Lund University Diabetes Centre, Malmö, Sweden; 4 Sahlgrenska Academy, Institute of Medicine, Department of Molecular and Clinical Medicine and Wallenberg Laboratory, University of Gothenburg, Gothenburg, Sweden; 5 Department of Cell and Organism Biology, Lund University, Lund, Sweden; 6 Innovation Center STB, Tczew, Poland; 7 Department of Clinical Biochemistry, Rigshospitalet, University of Copenhagen, Copenhagen, Denmark; 8 Diabetes and Endocrinology, Department of Clinical Sciences Malmö, Lund University Diabetes Centre, Malmö, Sweden; 9 Surgery, Department of Clinical Sciences Lund, Lund University, Lund, Sweden; 10 Molecular Metabolism, Department of Clinical Sciences Malmö, Lund University Diabetes Centre, Malmö, Sweden; 11 Centre for Analysis and Synthesis, Department of Chemistry, Lund University, Lund, Sweden; Western University of Health Sciences, UNITED STATES

## Abstract

**Background:**

A growing body of literature on Roux-en-Y gastric bypass surgery (RYGB) has generated inconclusive results on the mechanism underlying the beneficial effects on weight loss and glycaemia, partially due to the problems of designing clinical studies with the appropriate controls. Moreover, RYGB is only performed in obese individuals, in whom metabolism is perturbed and not completely understood.

**Methods:**

In an attempt to isolate the effects of RYGB and its effects on normal metabolism, we investigated the effect of RYGB in lean pigs, using sham-operated pair-fed pigs as controls. Two weeks post-surgery, pigs were subjected to an intravenous glucose tolerance test (IVGTT) and circulating metabolites, hormones and lipids measured. Bile acid composition was profiled after extraction from blood, faeces and the gallbladder.

**Results:**

A similar weight development in both groups of pigs validated our experimental model. Despite similar changes in fasting insulin, RYGB-pigs had lower fasting glucose levels. During an IVGTT RYGB-pigs had higher insulin and lower glucose levels. VLDL and IDL were lower in RYGB- than in sham-pigs. RYGB-pigs had increased levels of most amino acids, including branched-chain amino acids, but these were more efficiently suppressed by glucose. Levels of bile acids in the gallbladder were higher, whereas plasma and faecal bile acid levels were lower in RYGB- than in sham-pigs.

**Conclusion:**

In a lean model RYGB caused lower plasma lipid and bile acid levels, which were compensated for by increased plasma amino acids, suggesting a switch from lipid to protein metabolism during fasting in the immediate postoperative period.

## Introduction

Obesity increases at a pandemic rate. Consequently, the incidence of type 2 diabetes mellitus (T2DM) increases. For long, conservative treatments, *i*.*e*. diet- and exercise-interventions, have been the most common options to treat obesity and to reduce the risk of developing both obesity and T2DM [1]. Accordingly, dietary changes have been fundamental in the treatment of T2DM, most often with the aim of reducing caloric intake [2]. More recently, one week of a strict low-calorie diet was shown to normalize glycaemia in obese T2DM patients [3]. Although the improvements in glycaemia were maintained as long as 12 weeks post intervention, most diet- and exercise-interventions eventually fail to sustain long-term weight-loss [4].

Another, more invasive approach to induce weight-loss is bariatric surgery, which produces greater and more sustainable weight-loss and a higher rate of T2DM remission than life-style interventions [5]. Several different surgical techniques are available that reduce food intake and in some cases believed also to induce malabsorption [6]. Currently, Roux-en-Y gastric bypass surgery (RYGB) is the most common procedure, inducing both a reduction in food intake and to some extent nutrient malabsorption. RYGB has been shown to yield a higher degree of T2DM remission than most other available bariatric surgery procedures, both in the short (days) [7] and in the long term (4 years) [8]. The mechanism underlying T2DM remission is under debate. On one hand, it has been suggested that T2DM remission is solely due to reduced nutrient intake/malabsorption and weight-loss [9]. On the other hand, T2DM remission has been attributed to weight-loss-independent factors [8], such as altered circulating levels of gut hormones [10], particularly GLP-1 [11], changes in gut microbiota [12], gut nutrient sensing [13] and bile flow [14]. It has also been shown that plasma bile acid levels increase after RYGB in both the short [14] and the long term [15]. Besides their established role to improve digestion and lipid adsorption, bile acids also act on receptors, including the farnesoid-X-receptor (FXR) [16], enhance energy expenditure [17] and stimulate secretion of hormones such as GLP-1 [18]. However, the mechanism of bile acids in the improvement of glycaemia has become even more complex due to studies revealing that treatment with bile acid sequestrants, which increase faecal secretion of bile acids by disruption of their enterohepatic circulation, result in improved glycaemia in T2DM [19].

The impact of RYGB on metabolism in obese humans has been widely studied. However, the metabolic perturbations associated with obesity are only partially understood, potentially obscuring the mechanistic understanding of RYGB. We hypothesize that a clearer understanding of the mechanism may be achieved by studying the impact of RYGB under normal and healthy conditions. As it is unfeasible and unethical to perform such studies in humans, we used a lean porcine model [20], showing a greater resemblance to human physiology than rodents [21]. Pigs subjected to RYGB were compared with weight-matched, sham-operated, pair-fed control pigs, which allowed us to compensate for surgery-related stress, weight loss and nutrient intake. Although matching for nutrient intake is possible in human studies [9, 22], matching for confounding effects elicited by surgery per se is not feasible in humans.

## Materials and methods

### Animals

Experiments were performed with the approval of the Animal Ethics Committee of Lund University, Sweden (M128-12).

Weight-matched male juvenile landrace pigs [20] weighing 26±2.5 kg at the start of the study, were randomly selected from the University herd at Odarslöv research farm (Swedish Agricultural University, Alnarp, Sweden), transported to the animal facilities at the Department of Cell and Organism Biology (Lund University, Lund, Sweden) and then kept in individual pens with wood chips as bedding material. All pens were equipped with a dry feeding trough, a drinking nipple and a constant heating lamp (150 W). Prior to surgery, pigs were fed a standard diet (VÄXTILL®, Lantmännen, Sweden), at 4% of their body weight (approximately 2900 kcal) per day. The pens were cleaned and bedding material was replaced every day.

### Roux-en-Y gastric bypass surgery and postsurgical management

**Fig 1:**
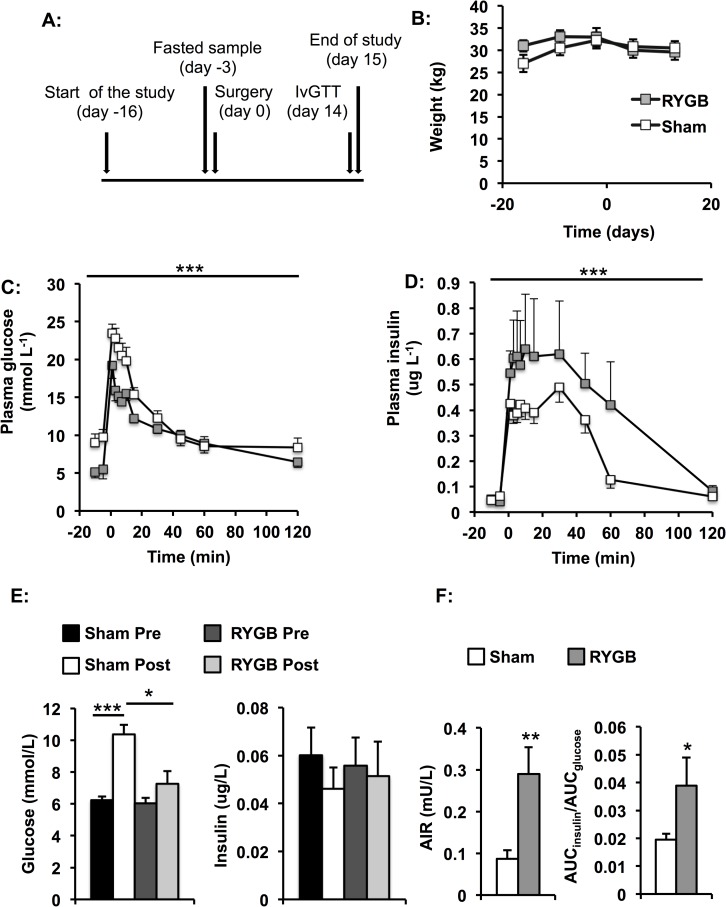
Changes in weight, insulin and glucose profiles after RYGB and sham-surgery. A: Time-line for the study. B: Weight developed similarly in the two groups of animals. C: Insulin levels were increased and D: glucose levels decreased in RYGB- compared with sham-pigs. E: Fasting glucose was higher in sham-pigs whereas insulin did not differ between the groups. F: RYGB-pigs had a higher acute insulin response (AIR) and AUC_insulin_/AUC_glucose_-ratio compared with the sham-pigs. Data are expressed as mean±SEM for n = 5 for RYGB and n = 8 for sham. Statistical differences were assessed by the two-way ANOVA in panels B, C and D, a one-way ANOVA in panel E, and the two-tailed heteroscedastic Student's t-test in panel F. *p<0.05, **p<0.01, ***p<0.001

Pigs were chosen as a model as they are omnivorous, have a GI-tract similar to the human and similar eating habits as humans. A time-line for the study is shown in Fig 1A.

Animals were subjected to either a standard limb RYGB (n = 9) or sham-surgery (n = 8) through upper-midline incisions under general halothane anaesthesia at the animal facilities at the Department of Cellular and Organism Biology as previously described in detail [20]. Briefly, a 12–15 ml gastric pouch was constructed by dividing the stomach 3 cm from the gastroesophageal transition. The jejunum was divided 60 cm distal to the duodenojejunal junction. A standard gastrojejunostomy and an enteroenterostomy were performed. Sham-surgery was performed in control animals, with gentle manipulation of the bowel but without transections.

Pigs were closely monitored, and treated prophylactically with ampicillin and buprenorphine until 3 days after surgery, as previously described in detail [20]. They were given three meals per day of low-calorie diet (250 ml Modifast, Stocksund, Sweden; 220 kcal, 25E% protein, 52E% carbohydrates, and 21E% fat) [20]. All pigs consumed all food provided. Thereby nutrient intake did not differ between pigs. Two RYGB-pigs died shortly after surgery; RYGB survival rate was 80%. One RYGB-pig died from ruptured staple line and another from post-operative swelling of an anastomosis. This number was within the anticipated range. At the end of the study, all pigs were euthanized by an overdose of anaesthesia (halothane) and injected with potassium chloride.

### Blood sampling and intravenous glucose tolerance test

Two weeks after arrival to the animal facilities, three days preoperatively, baseline blood samples were obtained after an overnight fast. Two weeks post-surgery, pigs were subjected to a frequently sampled IVGTT. The IVGTT was performed after an overnight fast using a glucose bolus of 500 mg ml^-1^ (1 g kg^-1^). Blood was collected in pre-chilled EDTA tubes at indicated time-points. Tubes were kept on ice until centrifugation (1500xg, 15 min, 4°C) and plasma stored at -80°C until analysis. One catheter in one of the RYGB-pigs was clogged, whereby only one fasted sample could be collected in this animal. This sample was used for analysis of bile acids. Due to problems with the catheter in another RYGB-pig, some data points are lacking. For two of the sham pigs, some samples were consumed prior to analysis of metabolite profiles.

### Glucose, insulin and cholecystokinin analysis

Plasma glucose was measured by the Infinity Glucose Oxidase kit (ThermoScientific, Lexington, MA) and insulin using a porcine ELISA (Mercodia, Uppsala, Sweden), according to the manufacturers' instructions. Cholecystokinin (CCK) was assayed by RIA [23]. HOMA-IR was calculated as basal insulin (μIU ml^-1^) x basal glucose (mmol L^-1^)/22.5, HOMA-β as (20 x basal insulin)/(basal glucose-3.5), and the acute insulin response (AIR) as mean insulin secretion for the first 10 min of the IVGTT. Insulin sensitivity (cSi) was estimated as the 0.276 x (glucose elimination rate / insulin AUC / 50), using data collected between 10 and 60 min of the IVGTT [24].

### Analysis of lipoprotein fractions and sub-fractions

Lipoprotein fractions and sub-fractions were separated based on size using Quantimetrix Lipoprint® system (Quantimetrix Corporation, Redondo Beach, CA) according to the manufacturers' instructions. Briefly, 25 μl of fasted serum collected prior to the IVGTT were mixed with 200 μl of loading gel and added to precast 3% polyacrylamide gel tubes. The mixture was subsequently polymerized at room temperature for 35 min and samples electrophoresed for 1h (3 mA per gel tube). Levels of cholesterol in each sub-fraction were calculated using the Lipoware Clinical analysis software (Quantimetrix Corporation). Total serum cholesterol was measured by a colorimetric assay (InfinityTM-Cholesterol; Thermo Scientific, Middletown, VA) as described previously [25].

### Metabolite profiling

Metabolite profiling was performed on samples acquired at baseline, pre- and post-surgery, and at 7 and 120 minutes during the IVGTT. Metabolites were analysed by GC/MS, as previously described in detail [26].

### Bile acid analysis

Samples for analyses of bile acids in plasma, faeces and bile were taken at sacrifice, after an overnight fast (15 hours), and 15 days post-surgery. Bile acids in plasma were extracted as previously described in detail [27]. Bile acids from faeces were extracted after homogenizing in 2 ml propylene tubes with zirconium oxide beads. Approximately 50 mg of faeces was placed in the tube and 500 μl methanol containing 2.5 μmol l^-1^ of internal standards was added. The sample was homogenized using a TissueLyser II (Qiagen, Hilden, Germany) at 25 Hz for 10 minutes. The homogenate was then centrifuged at 20 000xg for 10 minutes and 20 μl of the supernatant was diluted to 1000 μl with water/methanol (1/1; v/v). Bile acids from 50 μl of bile taken from the gallbladder using a syringe and needle were extracted by adding 500 μl methanol. After mixing and centrifugation the supernatant was diluted 1000-fold with water/methanol (1/1; v/v). Internal standards were added after the dilution. Extracted bile acids were analysed as previously described in detail [27].

### Statistical analysis

Hormone, metabolite and bile acid data are presented as mean ± SEM. Lipoprint and metabolite profiling data were mean centred and unit variance scaled and analysed by orthogonal projections to latent structures discriminant analysis (OPLS-DA) in Simca P+ 12 (Umetrics, Umeå, Sweden). AUC was calculated in GraphPad Prism 5 (GraphPad Software, San Diego, CA). Unpaired two-tailed Student’s t-test was used to assess statistical differences between RYGB- and sham-pigs and paired two-tailed Student’s t-test to assess differences between pre- and post-surgical levels in the two different groups of animals. ANOVA was used when several groups were compared. P-values of < 0.05 were considered significant.

## Results

### IVGTT-elicited plasma glucose levels are lower and insulin levels higher in RYGB- compared with sham-pigs

Body weight did not differ significantly between groups at any time of the study (Fig 1B, S1 Table). At the end of the study, RYGB-pigs weighed 28.9±1.9 kg and sham-pigs 29.6±1.3 kg. Insulin levels were higher (p<0.001) and glucose levels lower (p<0.001) during an IVGTT in the RYGB- compared with the sham-pigs (Fig 1C and 1D, S2 Table). Fasting glucose concentrations were 29% lower (p<0.01) in RYGB- compared with sham pigs, whereas fasting insulin did not differ between groups (Fig 1E). However, this between group difference was largely driven by a more pronounced increase in plasma glucose in the sham-pigs, which is likely a result from differences in diets and feeding protocols between pre- and post surgical care. Glucose elimination rate and peak glucose levels did not differ significantly between RYGB- and sham-pigs. Measures of β-cell secretory response, *i*.*e*. AIR and AUC_insulin_/AUC_glucose_-ratio, were 3.3-fold (p<0.01) and 2-fold (p<0.05), respectively, greater in RYGB- compared with sham-pigs (Fig 1F). HOMA-IR (RYGB, pre 0.016±0.0039, post 0.017±0.0049; sham, pre 0.017±0.0033, post 0.020±0.0038), cSi (RYGB, post 1.16±0.62 10^−4^ min^-1^ [μU/ml]^-1^; sham, post 3.23±0.83 10^−4^ min^-1^ [μU/ml]^-1^) HOMA-β (RYGB, pre 0.44±0.062, post 0.35±0.17; sham, pre 0.47±0.10, post 0.16±0.045) and plasma CCK levels (RYGB, post 0.93±0.10 pmol/L; sham, post 0.89±0.19 pmol/L) did not differ between the two groups.

### Reduced levels of VLDL-remnants and IDL particles in RYGB- compared with sham-pigs

**Fig 2:**
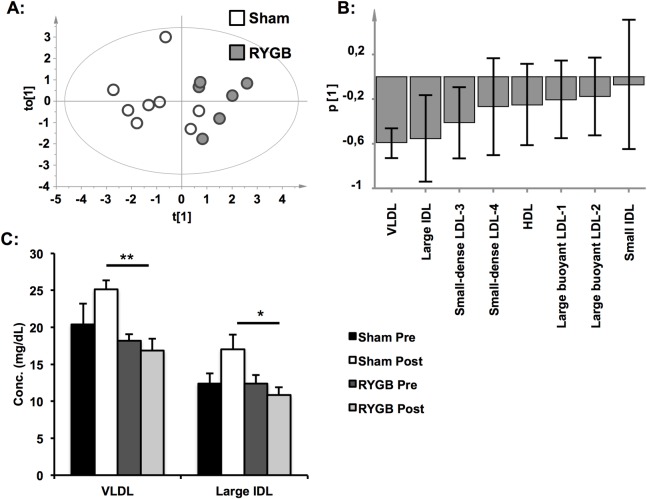
Lipoprotein subfractions after RYGB and sham-surgery. A: Lipoprotein data from RYGB- and sham-pigs were analysed by OPLS-DA to eliminate variation unrelated to the type of surgery. The score-scatter plot reveals that samples cluster depending on type of surgery. B: According to the loadings of the OPLS-DA model, with jack-knifed confidence intervals, levels of VLDL, large IDL and small-dense LDL-3 were lower after RYGB. C: Analysis of the raw data revealed lower levels of VLDL and large IDL cholesterol in RYGB-pigs. Data is expressed as mean±SEM for n = 6 for RYGB and n = 8 for sham in panel **(c)**. Statistical differences were assessed by ANOVA followed by Tukey's test *post hoc*. *p<0.05, **p<0.01. R^2^(Y) = 0.64 and Q^2^(Y) = 0.43 for the OPLS-DA model.

Differences in fasting lipoprotein content and composition between RYGB- and sham-pigs were analysed by OPLS-DA (S3 Table). OPLS-DA has the ability to separate predictive variation from variation that is uncorrelated to the investigated variable, thereby filtering out unwanted variation in the data. Here, OPLS-DA was used to exclude variation in the data unrelated to the surgical procedure, thereby focusing the analysis on alterations in the lipoprotein profile caused by RYGB. The score-scatter plot (Fig 2A) revealed that samples clustered according to whether they were derived from a RYGB- or a sham-pig. Differences in parameters underlying the observed clustering are visualized in the loading plot (Fig 2B). Hence, most lipoprotein fractions were lower in RYGB- compared with sham-pigs. Results from the OPLS-DA analysis were confirmed by univariate statistics, revealing lower levels of VLDL (-33%, p<0.01) and IDL-1 (large IDL; -36%, p<0.05) in RYGB- compared with sham-pigs (Fig 2C).

### Fasting amino acid levels increase after RYGB

Next, we analysed changes in fasting metabolite levels before and after RYGB or sham-surgery (S4 Table) by OPLS-DA; one model was calculated for the RYGB-pigs and one for the sham-pigs with sampling time, before or after surgery, as response (S1 Fig). The loadings from these models, scaled as correlations, were subsequently combined in a shared and unique structures (SUS) plot [28] (S1 Fig). From this plot, alterations in fasting metabolite levels elicited by either RYGB or sham-surgery could be identified. Interestingly, levels of several metabolites changed in opposite directions after RYGB and after sham-surgery. Thus, levels of the majority of amino acids increased after RYGB, whereas levels of all but four amino acids were decreased after sham-surgery. Furthermore, levels of NEFAs were unaffected by RYGB, but increased after sham-surgery. Notably, the branched-chain amino acids (BCAAs) valine, leucine and isoleucine decreased only after sham-surgery. Decreased levels of cysteine were seen after both RYGB and sham-surgery.

**Fig 3:**
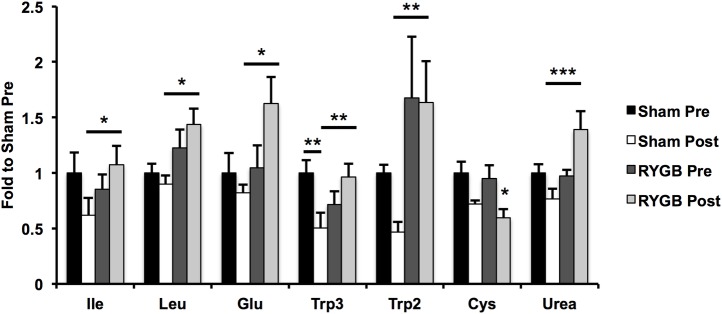
Alterations in fasted metabolite levels after RYGB and sham-surgery. Levels of multiple amino acids are higher in RYGB-pigs than in sham-pigs. Data are presented as mean±SEM for n = 6 for RYGB and n = 8 for sham. Statistical differences were assessed by the ANOVA, with pigs nested in surgical procedure, using Tukey's test *post hoc*. *p<0.05, **p<0.01, ***p<0.001.

Finally, we validated findings from the OPLS-DA models using ANOVA (Fig 3). Four amino acids, isoleucine (1.7-fold, p<0.05), leucine (1.6-fold, p<0.05), glutamate (2.0-fold, p<0.05) and tryptophan (2.7-fold, p<0.01) were higher after RYGB than after sham-surgery (Fig 3). In addition to the amino acids, urea (1.8-fold, p<0.05) was higher after RYGB than after sham-surgery. Cysteine levels decreased significantly only after RYGB (-37%, p<0.05). These changes were driven by both a decrease in levels in sham-pigs and increased levels in RYGB-pigs. Notably, there was an association between a larger weight loss and higher post-surgery fasting levels of the amino acids glutamate, histidine, lysine, leucine and tryptophan and the fatty acids C12:0, C14:0, C20:4, and C22:6 (p<0.05).

### Enhanced suppression of amino acid levels by glucose in RYGB-pigs

**Fig 4:**
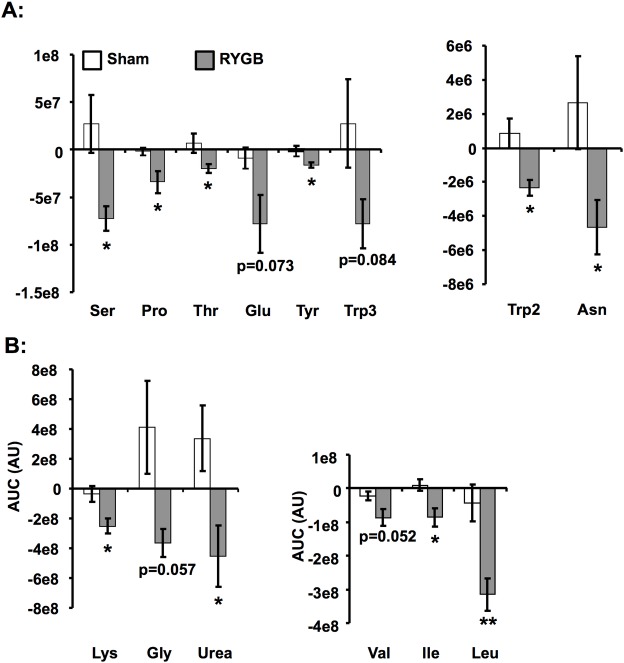
Alterations in metabolite levels elicited by an IVGTT in RYGB- and sham-operated pigs. Metabolite AUCs, calculated over fasted levels during the IVGTT after RYGB and sham-surgery, reveal a more pronounced glucose-elicited suppression of amino acids in RYGB-pigs compared with sham-pigs. Data is presented as mean±SEM for n = 6. Statistical differences were assessed by the two-tailed heteroscedastic Student's t-test. *p<0.05, **p<0.01. Abbreviations in supplementary material S1 Table.

We then investigated alterations in the metabolite profile elicited by administration of an intravenous glucose bolus. Calculation of AUCs for metabolites over fasting levels during the IVGTT (Fig 4, S4 Table) revealed that glucose-elicited suppression of amino acid levels was more efficient in RYGB-pigs than in sham-pigs. Hence, the AUCs of the BCAAs isoleucine (p<0.05), leucine (p<0.01) and valine (p = 0.052), as well as the amino acids lysine (p<0.05), serine (p<0.05), proline (p<0.05), threonine (p<0.05), tyrosine (p<0.05), tryptophan (p<0.05), and asparagine (p<0.05) were lower in RYGB-pigs than in sham-pigs. Also suppression of urea levels during the IVGTT was more pronounced in RYGB-pigs than in sham-pigs (p<0.05). The AUCs for threonine, tryptophan, asparagine, ornithine, creatinine (all p<0.05), an arginine product (p<0.01), and urea (p = 0.54) were associated with a higher glucose level at 120 min of the IVGTT. Hence, in the RYGB-pigs, with their lower glucose levels, amino acid levels were more effectively suppressed.

### RYGB causes increased gallbladder bile acids and reduced plasma and faecal bile acid levels

**Fig 5:**
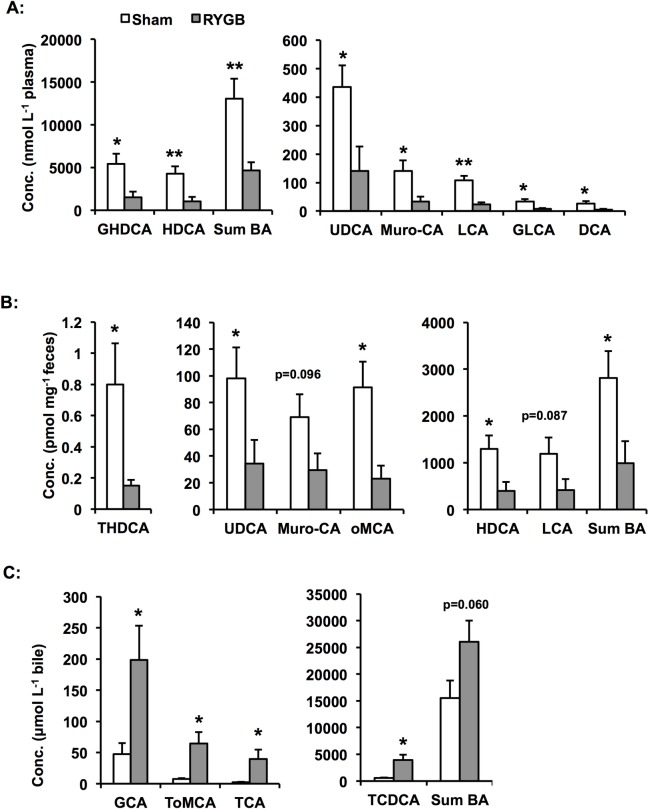
Faecal, circulating and gallbladder bile acid levels after RYGB and sham-surgery. A: Circulating levels of bile acids are reduced in RYGB-pigs compared with sham-pigs. B: Faecal bile acid levels are lower in RYGB-pigs compared with sham-pigs. C: Levels of bile acids are higher in bile from the gallbladder of RYGB-pigs compared with sham-pigs. Data is presented as mean±SEM for n = 7 for RYGB and n = 8 for sham. Statistical differences were assessed by the two-tailed heteroscedastic Student's t-test. *p<0.05, **p<0.01.

Our result revealed lower fasting lipid levels in RYGB-pigs compared with sham-pigs. To investigate whether this was related to changes in bile acid discharge we profiled fasting bile acids in plasma, faeces and in bile from the gallbladder (S5 Table). These analyses showed that RYGB-pigs had lower levels of all identified bile acids in plasma (-64% to -78%, p<0.05) and faeces (-57% to -81%, p<0.05), resulting in 81% (p<0.05) and 65% (p<0.05) lower total bile acid levels in plasma and faeces (Fig 5A and 5B, respectively). Total gallbladder bile acid levels trended to be higher in RYGB-pigs compared with sham-pigs (1.7-fold, p = 0.060) and the levels of all identified bile acids were 4.2- to 16-fold elevated (p<0.05) in the gallbladder bile of RYGB-pigs (Fig 5C). Gallbladder volume, estimated from total bile weight, did not differ between RYGB-pigs (37.7±6.1 g) and sham-pigs (29.6±2.2 g).

## Discussion

RYGB has been shown to cause remission of T2DM in the majority of patients [5, 8]. However, the mechanism underlying improved glucose metabolism has remained elusive. We chose to perform these experiments in a non-diabetic, non-obese pig model of RYGB rather than in an obese diabetic model. The purpose was to investigate the physiology of this procedure in the absence of confounding influence from T2DM and obesity and to enable pair-feeding for the elimination of differences in nutrient intake.

Both groups of pigs were weight stable during the test period. Hence, weight loss did not differ between RYGB-pigs and sham-pigs. However, due to large individual variation in weight between pigs the study is underpowered to detect small weight differences. Nevertheless, RYGB-pigs had lower fasting glucose, as well as higher insulin and lower glucose levels during an IVGTT compared with sham-pigs. In agreement with our previous study using this model [20] RYGB-pigs also displayed enhanced β-cell function. Insulin resistance and sensitivity, approximated by HOMA-IR and cSi, respectively, did not differ between groups. This observation is in concordance with a previous study on RYGB in lean Ossabaw miniature swine [29]. In the present study, we do not observe the expected improvement in insulin sensitivity after RYGB [30]. However, it has been shown that severe starvation reduces insulin sensitivity [31]. Hence, as the pigs used in the present study are lean, they may be more sensitive to starvation, as compared with the obese individuals that are usually subjected to RYGB.

A central aim of the present study was to extend findings of the effects of RYGB on glucose metabolism to other metabolic pathways starting with lipoprotein metabolism. We found lower levels of VLDL and large IDL in RYGB-pigs than in sham-pigs, confirming previous clinical studies that have reported lower circulating total and LDL cholesterol concentrations after RYGB [31]. Detailed investigations on different lipoprotein fractions have been lacking and a significant reduction in these major triglyceride-containing lipoproteins in fasted subjects have not been previously observed. Hypertriglyceridemia has long been recognized as an independent risk factor for cardiovascular disease, but it is the small VLDLs and IDLs, triglyceride-rich remnant lipoproteins that have emerged as the actual atherogenic agents [32]. IDLs have been shown to accumulate in atherosclerotic plaques *in vivo* [33] and play a role in the progression of atherosclerosis [34]. Taken together, our results show beneficial effects of RYGB on lipid metabolism, beyond those expected from weight-loss alone; possibly contributing to the reported reduced risk of cardiovascular disease [35].

Next, we examined the impact of RYGB on the plasma metabolite profile. Levels of several amino acids, including the BCAAs, and urea were higher in RYGB-pigs than in sham-pigs. Moreover, a large weight loss was associated with higher levels of most of these amino acids. Previous studies have shown BCAAs to decrease at one [22], three and six months [36] after RYGB. However, the impact of RYGB on BCAA levels is complex, as levels of these amino acids have been shown to associate with obesity and insulin resistance [37] as well as being reduced in response to a reduction in nutrient intake [38], but increased in response to starvation [39]. Moreover, BCAAs also decrease after non-operative weight loss interventions [38], although to a lesser extent than after RYGB [22, 38]. Hence, increased levels of amino acids observed in the RYGB-pigs may be due to starvation, which is in line with the observed trend towards lower insulin sensitivity in these pigs. Levels of amino acids and urea were also more efficiently suppressed by glucose in the RYGB-pigs. As opposed to the fasting levels, the improved suppression is likely mediated by insulin, the secretion of which is enhanced in response to glucose. As insulin sensitivity did not vary between groups, lower circulating lipid and glucose levels and higher amino acid and urea levels in RYGB-pigs compared with sham-pigs may suggest a more severe starvation in these pigs that, as opposed to previous studies in humans, were non-obese and had a normal metabolism at baseline.

Finally, we examined the impact of RYGB on bile acid levels. We found massively reduced levels of faecal and circulating bile acid levels in RYGB-pigs. Importantly, these alterations were associated with increased gallbladder bile acid levels in the RYGB-pigs, despite similar gallbladder volume as in the sham-pigs. These differences indicate that bile discharge may be disrupted in the RYGB-pigs, which in turn would impact on lipid emulsification and uptake. Opposite to what we observe here, circulating bile acid levels have previously been shown to be increased after RYGB in obese patients [14, 40], an effect that was attributed to enhanced reabsorption of bile in the terminal ileum [14] and changes in bile acid synthesis [40]. Despite this, malabsorption of lipids have been observed at six months, but not at one year, post RYGB [41], and malabsorption of both lipids and protein at 5 and 14 month after long-limb RYGB [42] where massive amounts of small intestine had been bypassed. Unfortunately, we lacked sufficient amounts of faeces to investigate faecal lipid content. Our data do however not support increased bile acid absorption, as this would be expected to result in elevated plasma bile acid levels.

We did not observe any differences in fasting CCK-levels, a major determinant of bile secretion, between RYGB- and sham-pigs, but cannot exclude an altered response to feeding, which could have been revealed by an OGTT or a mixed-meal test. Mixed-meal-elicited CCK secretion has been shown to be lower after RYGB compared with sleeve gastrectomy, which induce similar reduction in food intake, but have different impact on nutrient levels in the duodenum [43]. On the other hand, meal-elicited circulating CCK levels have also been shown to increase after RYGB [30]. The impact of RYGB on CCK and bile acids in lean humans is unknown.

There are some limitations to the study. Firstly, we cannot rule out that changes in metabolism are caused by altered insulinization of the liver and insulin clearance. Increased insulin clearance has previously been suggested to result in an underestimation of insulin levels following RYGB in humans [44]. Secondly, we cannot rule out an influence from alterations in the gut microbiota, which has emerged as an important regulator of host metabolism [45], and has been shown to be altered by RYGB [27]. Thirdly, differences in feeding protocols between pre and post-surgical care are likely to have impacted on the results. In fact, this is the case also with most clinical studies addressing the impact of RYGB on metabolism.

In summary, our findings explore the impact of RYGB in lean animals. Notably, reduced circulating and faecal bile acid levels, reduced circulating lipid levels and increased circulating amino acid and urea levels in RYGB-pigs suggest a potential impact of RYGB on macronutrient utilization. The findings differ to some extent from what has been found in clinical studies on RYGB for obesity. Whether our observed changes remain over time, possibly impacting metabolism in patients reaching normal weight is unknown.

## Supporting information

S1 Table Pig weight. (XLSX)

S2 Table Insulin and glucose levels during the IVGTT. (XLSX)

S3 Table Lipoprint data. (XLSX)

S4 Table Metabolite profiling data. (XLSX)

S5 Table Bile acid levels in blood plasma, bile and faeces. (XLSX)

S1 Fig Alterations in fasted metabolite levels after RYGB and sham-surgery. (PDF)
