# Supplementary material for: The impact of Roux-en-Y gastric bypass surgery on normal metabolism in a porcine model
Source: PLoS One. 2017 Mar 3;12(3):e0173137. doi: 10.1371/journal.pone.0173137 (PMC5336237; doi:10.1371/journal.pone.0173137)
Supplement: S1 Fig — (PDF) [file pone.0173137.s006.pdf]

S1 Figure

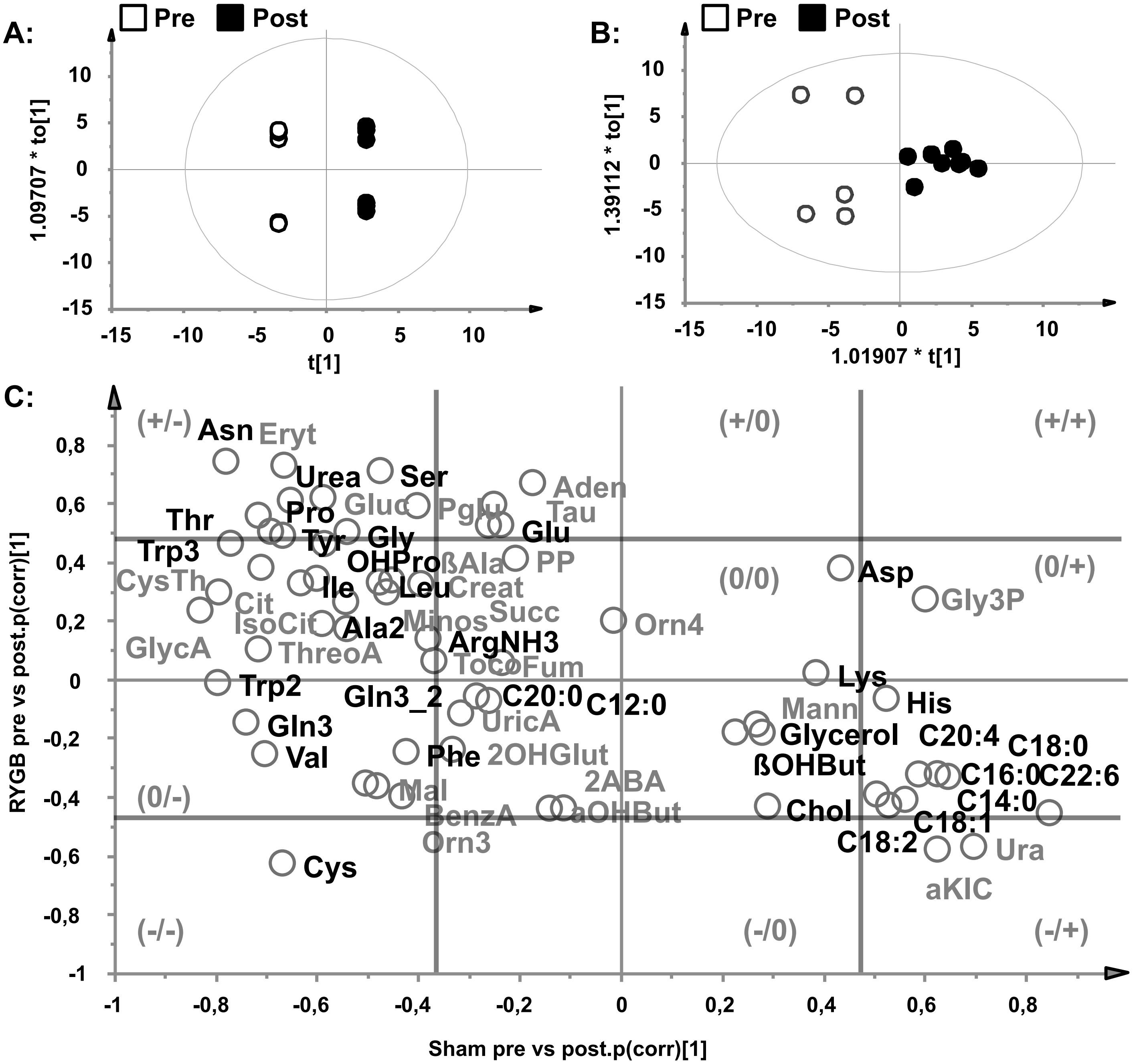

Alterations in fasted metabolite levels after RYGB- and sham-surgery. A: Classification of pre- and postoperative samples from the RYGB-operated pigs based on the metabolite profiles reveals that RYGB induces systematic alterations in the metabolite profiles. In this model the large number of measured variables (metabolites) have been reduced to a smaller number of independent variables. Each point in the plot represents a sample and the position of the point is determined by the magnitude of two of these variables. B: Classification of pair-fed sham-pigs as in panel A. C: Combination of the loadings derived from the two OPLS-DA models, scaled as correlations, in a SUS-plot reveals unique and shared alterations elicited by RYGB- and sham-surgery. Boarders for significant regulations have been derived from the corresponding loadings with jack-knifed confidence intervals. Unique, shared, inverse and insignificant regions are indicated as: (RYGB/Sham); +, up-regulated; -, down-regulated; 0, insignificant.
